# Differential Expression of Endocannabinoid Receptors in Lesional and Non-Lesional Skin of Psoriasis Patients: Insights Into Pathogenesis and Potential Therapeutic Targets

**DOI:** 10.1177/12034754251355199

**Published:** 2025-07-26

**Authors:** Jaime N. Turk, Mark G. Kirchhof

**Affiliations:** 1 Department of Medicine, University of Ottawa, Ottawa, ON, Canada; 2 Dermatology Division, Department of Medicine, University of Ottawa and The Ottawa Hospital, Ottawa, ON, Canada

**Keywords:** psoriasis, endocannabinoid system, gene expression, *Cannabis*

## Abstract

**Background:**

Psoriasis is a chronic, immune-mediated inflammatory skin disease with a complex etiology involving genetics, environmental triggers, and immune dysregulation. Research suggests that the endocannabinoid system (ECS) is involved in inflammation and skin homeostasis, prompting interest in its involvement in the pathogenesis of psoriasis.

**Objectives:**

This study was designed to investigate the expression of cannabinoid receptors and signaling channels in psoriatic-affected tissue (lesional), unaffected tissue (non-lesional), and healthy control subjects.

**Methods:**

Data were extracted using bulk RNA sequencing data from the Gene Expression Omnibus public database. Differential gene expression analysis was performed to determine changes in cannabinoid receptor expression between psoriatic lesional skin, non-lesional skin, and healthy controls.

**Results:**

We found that in psoriatic lesional skin, GPR12, PPARG, TRPV4, PPARA, and HTR1A were significantly downregulated, while CNR2, TRPA1, TRPV3, PPARD, GPR18, ADORA2A, HTR3B, and HTR3A were notably upregulated compared to healthy controls. In addition, TRPV4, PPARG, PPARA, and GPR12 were markedly downregulated in psoriatic lesional skin compared to non-lesional skin, while PPARD, HTR3A, HTR3B, GPR18, TRPV3, TRPA1, CNR2, and ADORA2A showed significant upregulation. There were no significantly upregulated or downregulated endocannabinoid genes in the non-lesional to healthy control analysis.

**Conclusions:**

These findings provide new insights into the role of the ECS in psoriasis pathogenesis and highlight potential targets for further research or novel therapeutic interventions.

## Introduction

Psoriasis is a lifelong, immune-mediated, inflammatory skin disease that affects an estimated 125 million people worldwide. Its pathogenesis involves a disturbance in the dynamic “cross-talk” between epidermal keratinocytes and cutaneous immune cells.^1,2^ The molecular understanding of psoriasis is still in its infancy, despite significant advances in understanding T-cell signaling, necessitating the need for further research.^3^

One area of emerging interest is the involvement of the endocannabinoid system (ECS) in the pathogenesis of psoriasis. It is comprised of endogenous ligands (endocannabinoids), synthesizing and degrading enzymes, and cannabinoid receptors.^4^ These receptors are categorized broadly into classical receptors, ionotropic receptors, novel receptors, nuclear receptors, and neurotransmitter ligand-activated channel receptors (Table 1).^2^ The ECS is expressed in many tissues throughout the body, including the skin, where it moderates various physiological processes.

**Table 1.** Categories of Cannabinoid Receptors.

Classic	CB1R or CNR1, CB2R or CNR2
Ionotropic	TRPV1, TRPV2, TRPV3, TRPV4, TRPA1, TRPM8
Novel	GPR3, GPR6, GPR12, GPR18, GPR55, LPAR5, GPR119
Nuclear	PPARA, PPARG, PPARD
Neurotransmitter ligand-activated channel	HTR1A, HTR2A, HTR3A, HTR3B, HTR3C, HTR3D, a2-AR (adenosine A2A receptor, also known as ADORA2A)

Previous studies suggest the ECS influences skin homeostasis,^5^ cell growth, and survival,^6^ and cutaneous immune and inflammatory processes.^7,8^ A study by Ständer et al observed abundant CB1/CB2 immunoreactivity in cutaneous nerve fiber bundles, mast cells, and epidermal keratinocytes.^9^ Another study by Özcan et al showed increased expression of TRPV1, an ionotropic receptor, in peripheral blood mononuclear cells of patients with psoriasis compared to healthy controls.^10^ Gene expression of PPARY, a nuclear receptor, was found to be downregulated in psoriasis plaques, while expression of PPARD was found to be upregulated in psoriasis plaques.^11,12^ However, a thorough comparison of all these receptors in lesional, non-lesional, and healthy controls has not been undertaken.

This study aims to investigate the expression levels of classical, ionotropic, novel, and nuclear cannabinoid receptors in lesional skin, non-lesional skin, and healthy control skin. Through quantification of the fold change in receptor expression among these groups, we seek to further elucidate the role of the ECS in psoriasis pathogenesis.

## Materials and Methods

### Data Extraction

RNA-seq sample expression data from Gene Expression Omnibus (GEO) series: GSE121212, GSE142582, GSE63980, GSE107871, GSE230200, and GSE193309 were obtained from the GEO database. A total of 402 samples were available, including skin samples from normal healthy donors (n = 235), lesional skin biopsy samples from psoriasis patients (n = 110), and non-lesional samples from psoriasis patients (n = 57).

### Data Processing and Differentially Expressed Gene Analysis

GEO series containing bulk RNA sequencing studies were identified, and associated Sequence Read Archives files were downloaded.^13^ By means of R version 4.3.2 (R Foundation for Statistical Computing)﻿, the samples were processed using the nf-core/rnaseq pipeline to obtain Salmon pseudocounts and quality metrics.^14^ Based on quality metrics from the rnaseq pipeline, samples were identified as poor quality and excluded from the analysis if they: contained <15 M reads after quality filtering and adapter trimming, if fewer than 70% of reads were uniquely mapped by Salmon to the transcriptome, and if they failed the FASTQC post quality filtering and adapter trimming GC content filter.

Salmon pseudocounts for each sample passing the above quality filtering were loaded from the pipeline output and then imported into DESeq2.^15^ Dimensionality reduction was performed using a principal component analysis (PCA). The fold change analysis was performed between the various groups: “lesional versus non-lesional,” “non-lesional versus healthy,” and “lesional versus healthy.” Fold change shrinkage was used to reduce estimated fold changes when uncertainty was high using the apeglm method. Volcano plots were generated for each, denoting the expression of genes, and the genes of interest were labeled accordingly.^16^ Significance of fold change of target genes of interest was identified.

To determine general trends in gene expression across samples (single or paired-end sequencing), disease condition, and GEO series, PCA plots were generated (Figures S1–S3). Hierarchical clustering was calculated using Euclidean distance between variance-stabilized normalized log count values for all transcripts (Figure S4).

### Adjustment for Sex

The gene XIST is highly expressed in females but has very low or zero expression in males. We annotated the sex of patient samples based on XIST expression, which allowed us to annotate samples based on predicted sex and correct for differences between conditions that are sex independent in the subsequent analyses.

## Results

### Comparison of Cannabinoid Receptors on Psoriatic Lesional Skin to Healthy Control Skin

**Figure 1.**
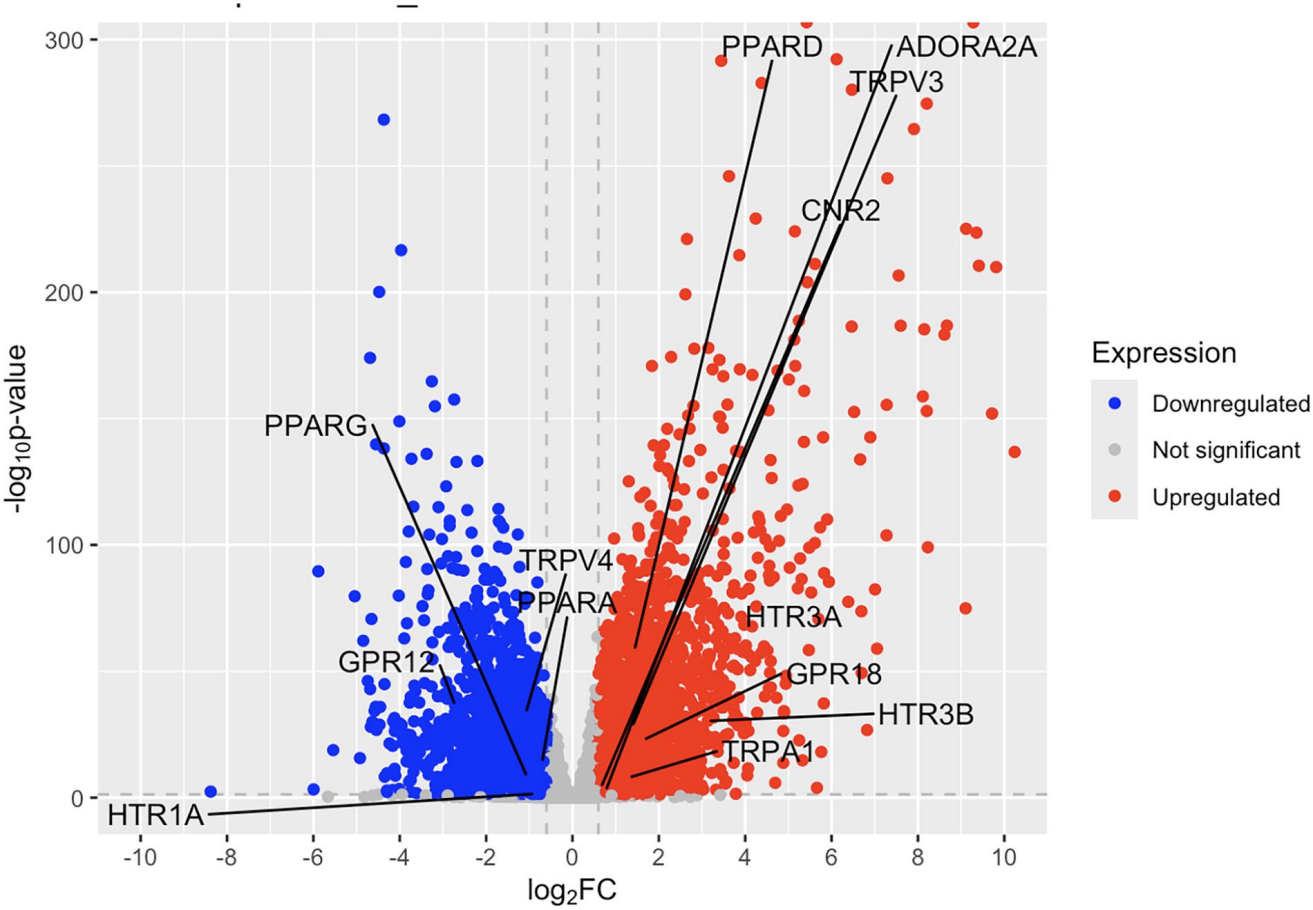
Volcano plot of fold change in psoriatic lesional skin compared to healthy controls.

In psoriatic lesional skin, GPR12, PPARG, TRPV4, PPARA, and HTR1A are significantly downregulated, while CNR2, TRPA1, TRPV3, PPARD, GPR18, ADORA2A, HTR3B, and HTR3A are notably upregulated compared to healthy controls (Figure 1 and Table S1). The HTR3A and HTR3B genes were the most upregulated genes, while GPR12 was the most downregulated gene. The CNR2 gene, which is one of the classic cannabinoid receptors, was upregulated with a log2 fold change of 0.661.

### Comparison of Cannabinoid Receptors on Psoriatic Non-Lesional Skin to Healthy Controls

**Figure 2.**
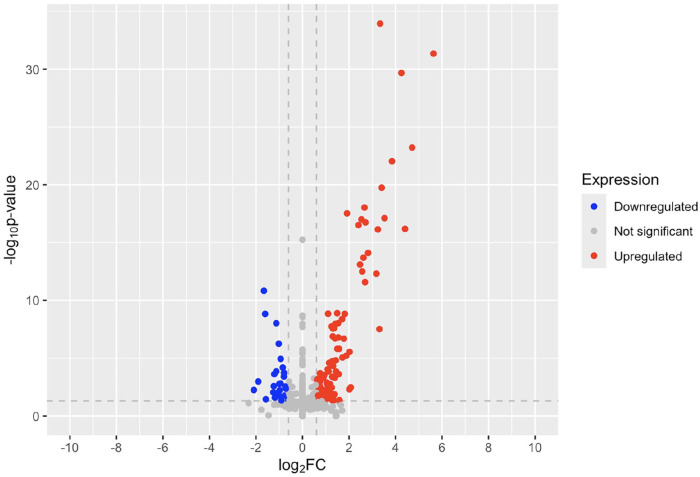
Volcano plot of fold change in psoriatic non-lesional skin compared to healthy controls.

There were no significantly upregulated or downregulated genes of interest in the psoriatic non-lesional to healthy control analysis (Figure 2). The volcano plot shows some gene expression differences between non-lesional and healthy skin, but none of the cannabinoid receptor genes were differentially expressed.

### Comparison of Cannabinoid Receptors on Psoriatic Lesional Skin to Non-Lesional Skin

**Figure 3.**
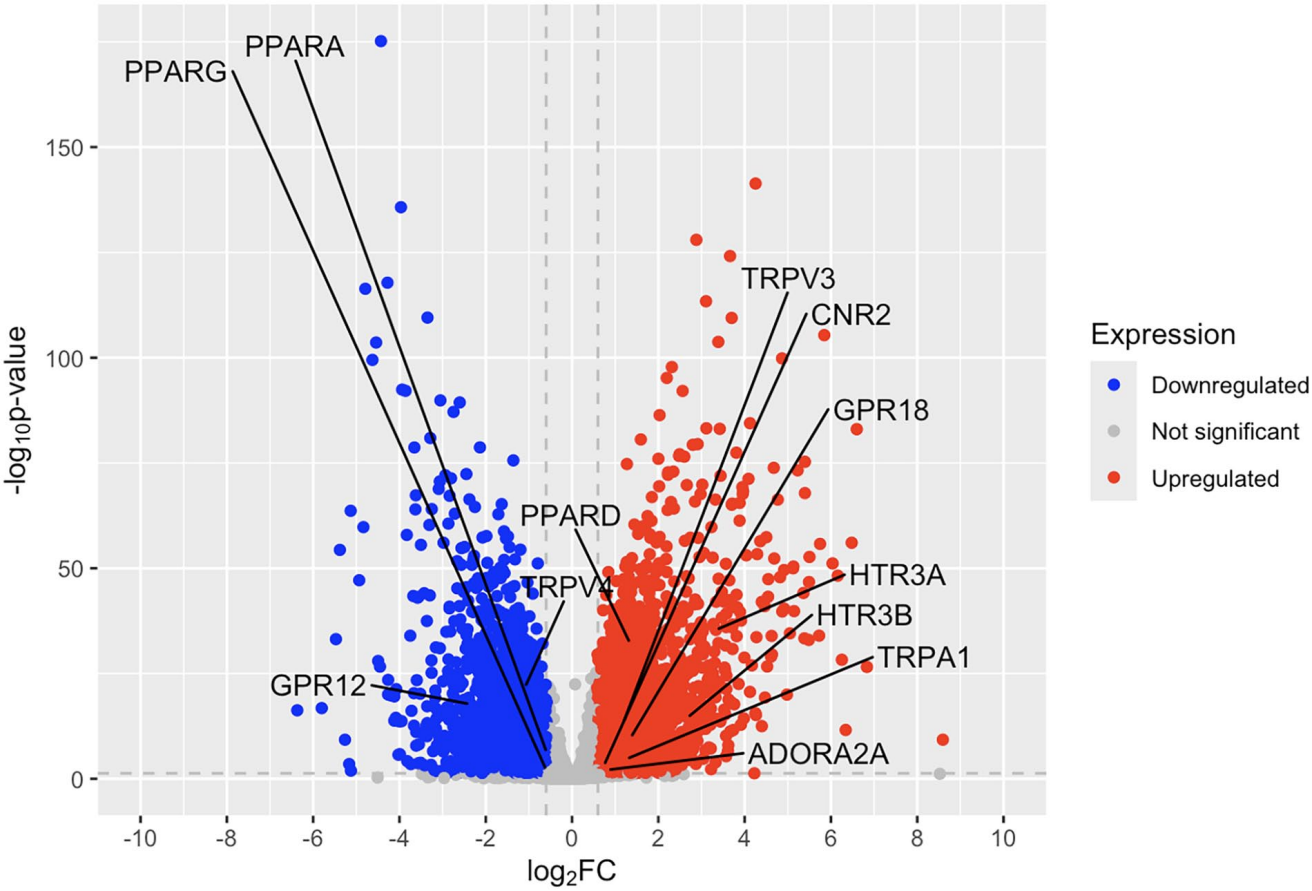
Volcano plot of fold change of psoriatic lesional skin compared to non-lesional skin.

In patients with psoriasis, TRPV4, PPARG, PPARA, and GPR12 are significantly downregulated in lesional skin compared to non-lesional skin, while PPARD, HTR3A, HTR3B, GPR18, TRPV3, TRPA1, CNR2, and ADORA2A show significant upregulation (Figure 3 and Table S2). As was seen in the lesional skin, HTR3A and HTR3B were the most highly upregulated genes compared to non-lesional skin. Like the lesional skin gene expression, GPR12 was the most downregulated gene with a log2 fold change of −2.36. The CNR2 gene, which is one of the classic cannabinoid receptors, was also upregulated with a log2 fold change of 0.739.

## Discussion

The findings from this study highlight the complex role of the ECS in the pathogenesis of psoriasis. The differential expression of various cannabinoid receptors and related genes in psoriatic non-lesional and lesional skin compared to healthy controls gives insights into their contribution to psoriasis pathogenesis (Table S3). It also highlights the potential therapeutic role of these receptors.

In both psoriasis lesional to healthy and lesional to non-lesional comparison, there was significant downregulation of TRPV4, PPARG, PPARA, and GPR12. TRPV4 is an ionotropic receptor that plays a significant role in skin barrier integrity, homeostasis, differentiation, nociception, and itch.^17^ Mouse models have shown that TRPV4-knockout mice had reduced epidermal thickness compared to wild-type mice, showcasing the potential protective effect of TRPV4 in reducing epidermal hyperproliferation in psoriatic lesions.^18^ Similarly, the nuclear receptors PPARG and PPARA play critical roles in regulating inflammation, lipid metabolism, and keratinocyte differentiation.^19^ The downregulation of these receptors suggests a disrupted lipid metabolism and a compromised anti-inflammatory response, which could exacerbate inflammation and contribute to the hyperproliferation of keratinocytes as seen in psoriasis.^20^ In addition, Sobolev et al found that PPARG weakens the expression of genes that contribute to the development of psoriatic lesions, which corroborates our findings.^11^ GPR12 is a novel receptor, and its role in skin disease remains largely unknown.

Unlike the receptors above, HTR1A was downregulated only in the psoriasis lesional compared to the healthy control. HTRA1 is a serine protease that plays a role in various diseases, including osteoarthritis and macular degeneration, by modulating the extracellular matrix.^21^

There was significant upregulation of PPARD, HTR3A, HTR3B, GPR18, TRPA1, TRPV3, CBR2/CNR2, and ADORA2A in both the psoriasis lesional to healthy and lesional to non-lesional comparison. PPARD has been previously shown to be upregulated in psoriasis, as its activation enhances the proliferation of keratinocytes.^22,23^

HTR3A and HTR3B are both subunits of the serotonin receptor (5-HT3). Serotonin (5-HT) is also a main factor in depression, which is one of the main comorbidities of psoriasis. Younes and Bakry found that serotonin expression was significantly higher in lesional biopsies from patients with chronic plaque psoriasis compared to controls.^24^ We found that serotonin receptor expression was elevated in psoriatic lesional skin compared to both non-lesional and healthy skin. This finding could potentially implicate the use of systemic serotonergic drugs in the treatment of psoriasis. A population-based cohort study by Thorslund et al revealed that the use of selective serotonin reuptake inhibitors (SSRIs) in patients with psoriasis was associated with a decreased need for systemic psoriasis treatment.^25^ While this study suggests a possible therapeutic effect of SSRIs, the exact mechanism remains unclear and could involve both anti-inflammatory properties and mood-related benefits that reduce scratching and trauma.

PPARD is a nuclear receptor known to regulate epithelial differentiation and wound healing. Romanowska et al concluded that activation of PPARD in the epidermis was sufficient to trigger inflammatory changes and immune activation characteristics of psoriasis.^26^ We found that PPARD expression is elevated in psoriatic lesional tissue compared to both non-lesional tissue and healthy controls.

TRPV3, an ionotropic receptor that is highly expressed in keratinocytes, was shown to be significantly increased in the epidermis of psoriatic lesional skin relative to adjacent non-lesional skin.^27^ Our study demonstrated that TRPV3 is not only upregulated in lesional skin relative to non-lesional skin but also elevated compared to healthy controls. A recent study on the role of TRPA1 in psoriasis found increased expression in psoriatic skin lesions.^28^ Paradoxically, psoriasiform dermatitis was significantly enhanced in TRPA1 knockout mice and with the treatment of a TRPA1 agonist.^28^

The Adenosine A2A receptor ADORA2A was upregulated in psoriatic lesional skin compared to non-lesional and healthy controls. This supports other findings that enhancing A2A receptor signaling through positive allosteric modulation can attenuate psoriasis-like dermatitis by reducing cytokine expression and improving clinical features in mouse models.^29^

CBR2 (also known as CN2R) was increased in lesional skin relative to non-lesional skin and healthy controls. CB2R is a classical cannabinoid receptor expressed in areas where there is active inflammation.^30^ Li et al found that CB2R expression levels were increased in mice with psoriasis. Moreover, treatment with a CB2R agonist significantly reduced inflammation and scratching bouts in the mice.^31^

This study offers a robust and novel contribution to the existing literature. The comprehensive analysis of multiple cannabinoid receptors from the same subjects offers a more complete picture of their implications in psoriasis pathogenesis. In addition, using public datasets from the GEO database allowed us to examine a large sample size (n = 402), which enhanced the statistical power and generalizability of findings.

Limitations include cellular heterogeneity, as bulk RNA sequencing measures average gene expression across all cell types in a sample, potentially masking receptor changes in specific cell populations. Further research involving single-cell RNA sequencing could provide additional insight. In addition, sample-specific properties such as demographics, disease severity, and treatment status can influence gene expression. However, because the datasets used in this study were drawn from multiple experiment series in the NCBI GEO, the metadata provided was inconsistent across studies, preventing a systematic analysis of these factors.

This study provides a comprehensive analysis of cannabinoid receptor expression in psoriatic lesional, non-lesional, and healthy skin. The observed differential expression patterns contribute to a deeper understanding of the ECS in psoriasis pathogenesis and offer new insights that could guide future research in exploring novel therapeutic strategies. By highlighting the potential role of cannabinoid receptors in immune regulation and skin inflammation, these findings underscore the need for further investigations into targeted cannabinoid-based therapies for psoriasis.

## Supplemental Material

sj-pdf-1-cms-10.1177_12034754251355199 – Supplemental material for Differential Expression of Endocannabinoid Receptors in Lesional and Non-Lesional Skin of Psoriasis Patients: Insights Into Pathogenesis and Potential Therapeutic Targets Supplemental material, sj-pdf-1-cms-10.1177_12034754251355199 for Differential Expression of Endocannabinoid Receptors in Lesional and Non-Lesional Skin of Psoriasis Patients: Insights Into Pathogenesis and Potential Therapeutic Targets by Jaime N. Turk and Mark G. Kirchhof in Journal of Cutaneous Medicine and Surgery

## References

[1] Bíró T Tóth BI Haskó G Paus R Pacher P . The endocannabinoid system of the skin in health and disease: novel perspectives and therapeutic opportunities. Trends Pharmacol Sci. 2009;30(8):411-420. doi:10.1016/j.tips.2009.05.004 19608284 PMC2757311

[2] Tóth KF Ádám D Bíró T Oláh A . Cannabinoid signaling in the skin: therapeutic potential of the “c(ut)annabinoid” system. Molecules. 2019;24(5):918. doi:10.3390/molecules24050918 30845666 PMC6429381

[3] Brembilla NC Senra L Boehncke WH . The IL-17 family of cytokines in psoriasis: IL-17A and beyond. Front Immunol. 2018;9:1682. doi:10.3389/fimmu.2018.01682 30127781 PMC6088173

[4] Navarrete F García-Gutiérrez MS Jurado-Barba R , et al. Endocannabinoid system components as potential biomarkers in psychiatry. Front Psychiatry. 2020;11:315. doi:10.3389/fpsyt.2020.00315 32395111 PMC7197485

[5] Caterina MJ . TRP channel cannabinoid receptors in skin sensation, homeostasis, and inflammation. ACS Chem Neurosci. 2014;5(11):1107-1116. doi:10.1021/cn5000919 24915599 PMC4240254

[6] Tóth BI Dobrosi N Dajnoki A , et al. Endocannabinoids modulate human epidermal keratinocyte proliferation and survival via the sequential engagement of cannabinoid receptor-1 and transient receptor potential vanilloid-1. J Invest Dermatol. 2011;131(5):1095-1104. doi:10.1038/jid.2010.421 21248768

[7] Río CD Millán E García V Appendino G DeMesa J Muñoz E . The endocannabinoid system of the skin. A potential approach for the treatment of skin disorders. Biochem Pharmacol. 2018;157:122-133. doi:10.1016/j.bcp.2018.08.022 30138623

[8] Karsak M Gaffal E Date R , et al. Attenuation of allergic contact dermatitis through the endocannabinoid system. Science. 2007;316(5830):1494-1497. doi:10.1126/science.1142265 17556587

[9] Ständer S Schmelz M Metze D Luger T Rukwied R . Distribution of cannabinoid receptor 1 (CB1) and 2 (CB2) on sensory nerve fibers and adnexal structures in human skin. J Dermatol Sci. 2005;38(3):177-188. doi:10.1016/j.jdermsci.2005.01.007 15927811

[10] Özcan S Gürel G Çakır M . Gene expression profiles of transient receptor potential (TRP) channels in the peripheral blood mononuclear cells of psoriasis patients. Hum Exp Toxicol. 2021;40(8):1234-1240. doi:10.1177/0960327121991911 33550865

[11] Sobolev V Nesterova A Soboleva A , et al. The model of *PPARγ*-downregulated signaling in psoriasis. PPAR Res. 2020;2020:1-11. doi:10.1155/2020/6529057 PMC7568796 33133175

[12] Hack K Reilly L Palmer C , et al. Skin-targeted inhibition of PPARβ/δ by selective antagonists to treat PPARβ/δ—mediated psoriasis-like skin disease in vivo. PLoS One. 2012;7(5):e37097. doi:10.1371/journal.pone.0037097 PMC3351437 22606335

[13] Ewels PA Peltzer A Fillinger S , et al. The nf-core framework for community-curated bioinformatics pipelines. Nat Biotechnol. 2020;38(3):276-278. doi:10.1038/s41587-020-0439-x 32055031

[14] R Core Team. R: A Language and Environment for Statistical Computing. R Foundation for Statistical Computing; 2023. https://www.R-project.org/ (Accessed April 14, 2025).

[15] Love MI Huber W Anders S . Moderated estimation of fold change and dispersion for RNA-seq data with DESeq2. Genome Biol. 2014;15(12):550. doi:10.1186/s13059-014-0550-8 25516281 PMC4302049

[16] Durinck S Spellman PT Birney E Huber W . Mapping identifiers for the integration of genomic datasets with the R/Bioconductor package biomaRt. Nat Protoc. 2009;4(8):1184-1191. doi:10.1038/nprot.2009.97 19617889 PMC3159387

[17] Moore C . The role of TRPV4 channels in cutaneous epithelia. Curr Top Membr. 2022;89:139-154. doi:10.1016/bs.ctm.2022.06.003 36210147 PMC9990182

[18] Amalia SN Baral H Fujiwara C , et al. TRPV4 regulates the development of psoriasis by controlling adenosine triphosphate expression in keratinocytes and the neuroimmune system. J Invest Dermatol. 2023;143(12):2356-2365.e5. doi:10.1016/j.jid.2023.05.009 37263487

[19] Schmuth M Jiang YJ Dubrac S Elias PM Feingold KR . Thematic review series: skin lipids. Peroxisome proliferator-activated receptors and liver X receptors in epidermal biology. J Lipid Res. 2008;49(3):499-509. doi:10.1194/jlr.R800001-JLR200 18182682

[20] Zhou X Chen Y Cui L Shi Y Guo C . Advances in the pathogenesis of psoriasis: from keratinocyte perspective. Cell Death Dis. 2022;13(1):81. doi:10.1038/s41419-022-04523-3 35075118 PMC8786887

[21] Yamawaki S Naitoh M Kubota H , et al. HtrA1 is specifically up-regulated in active keloid lesions and stimulates keloid development. Int J Mol Sci. 2018;19(5):1275. doi:10.3390/ijms19051275 29695130 PMC5983720

[22] Westergaard M Henningsen J Rasmussen S , et al. Expression and localization of peroxisome proliferator-activated receptors and nuclear factor κB in normal and lesional psoriatic skin. J Invest Dermatol. 2003;121(5):1104-1117. doi:10.1046/j.1523-1747.2003.12536.x 14708613

[23] Romanowska M Al Yacoub N Seidel H , et al. PPARδ enhances keratinocyte proliferation in psoriasis and induces heparin-binding EGF-like growth factor. J Invest Dermatol. 2008;128(1):110-124. doi:10.1038/sj.jid.5700943 17637826

[24] Younes SF Bakry OA . Immunohistochemical evaluation of role of serotonin in pathogenesis of psoriasis. J Clin Diagn Res. 2016;10(10):EC05-EC09. doi:10.7860/JCDR/2016/22692.8719 PMC5121680 27891342

[25] Thorslund K Svensson T Nordlind K Ekbom A Fored CM . Use of serotonin reuptake inhibitors in patients with psoriasis is associated with a decreased need for systemic psoriasis treatment: a population-based cohort study. J Intern Med. 2013;274(3):281-287. doi:10.1111/joim.12093 23711088

[26] Romanowska M Reilly L Palmer CNA Gustafsson MCU Foerster J . Activation of PPARβ/δ causes a psoriasis-like skin disease in vivo. PLoS One. 2010;5(3):e9701. doi:10.1371/journal.pone.0009701 PMC2838790 20300524

[27] Scott VE Patel H Wetter J , et al. 534 Defining a mechanistic link between TRPV3 activity and psoriasis through IL-1α and EGFR signaling pathways. J Invest Dermatol. 2016;136(5):S94. doi:10.1016/j.jid.2016.02.572

[28] Kemény Á Kodji X Horváth S , et al. TRPA1 acts in a protective manner in imiquimod-induced psoriasiform dermatitis in mice. J Invest Dermatol. 2018;138(8):1774-1784. doi:10.1016/j.jid.2018.02.040 29550417

[29] Welihinda A Ravikumar P Kaur M , et al. Positive allosteric modulation of A2AR alters immune cell responses and ameliorates psoriasis-like dermatitis in mice. J Invest Dermatol. 2022;142(3 Pt A):624-632.e6. doi:10.1016/j.jid.2021.07.174 34536482

[30] Bie B Wu J Foss JF Naguib M . An overview of the cannabinoid type 2 receptor system and its therapeutic potential. Curr Opin Anaesthesiol. 2018;31(4):407-414. doi:10.1097/ACO.0000000000000616 29794855 PMC6035094

[31] Li L Liu X Ge W , et al. CB2R deficiency exacerbates imiquimod-induced psoriasiform dermatitis and itch through the neuro-immune pathway. Front Pharmacol. 2022;13:790712. doi:10.3389/fphar.2022.790712 35173615 PMC8841964

