# Supplementary material for: Differential Expression of Endocannabinoid Receptors in Lesional and Non-Lesional Skin of Psoriasis Patients: Insights Into Pathogenesis and Potential Therapeutic Targets
Source: J Cutan Med Surg. 2025 Jul 26;30(1):56–61. doi: 10.1177/12034754251355199 (PMC12906604; doi:10.1177/12034754251355199)
Supplement: sj-pdf-1-cms-10.1177_12034754251355199 – Supplemental material for Differential Expression of Endocannabinoid Receptors in Lesional and Non-Lesional Skin of Psoriasis Patients: Insights Into Pathogenesis and Potential Therapeutic Targets [file sj-pdf-1-cms-10.1177_12034754251355199.pdf]

Supplemental Information

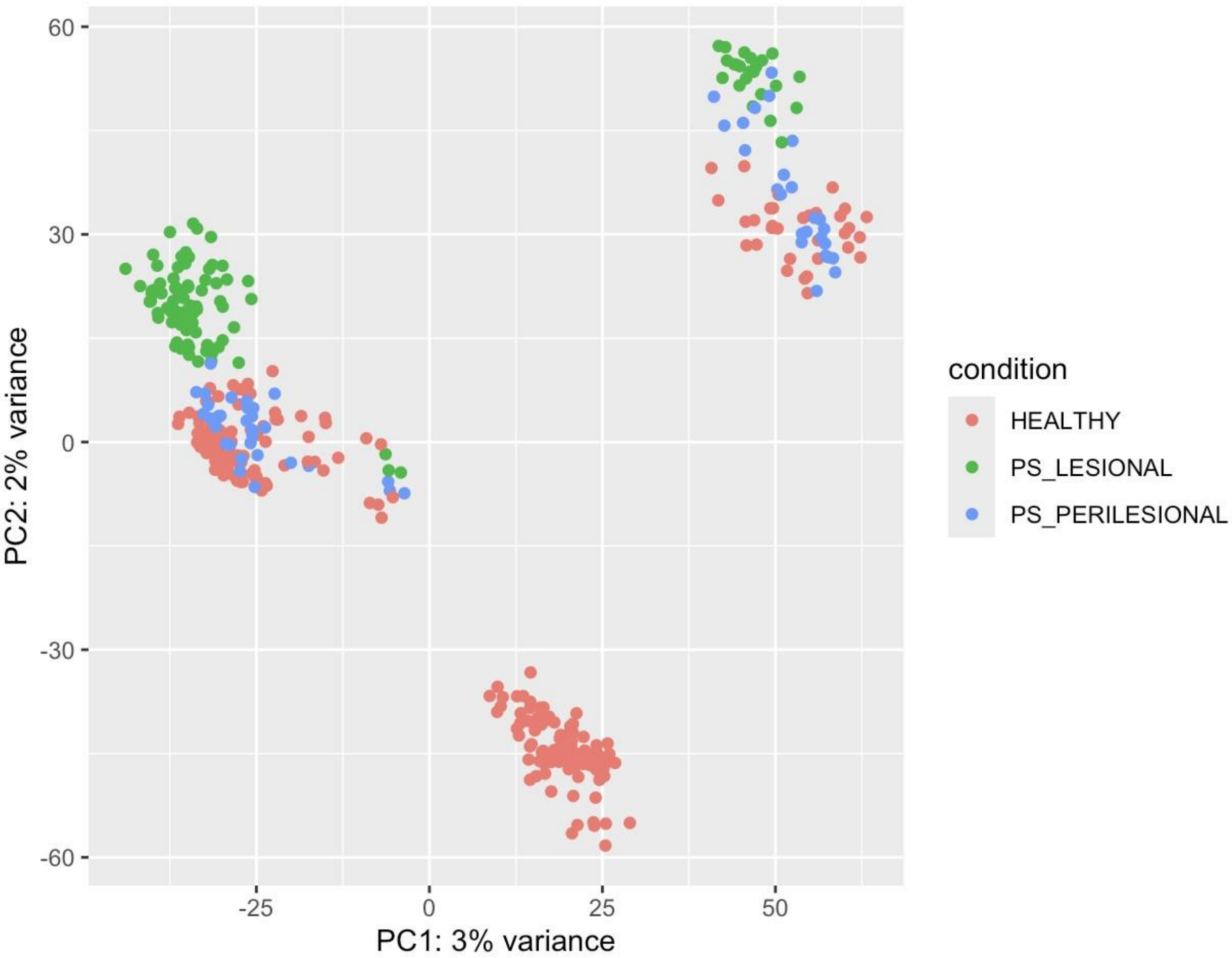

Figure S1: Principal component analysis (PCA) plot for disease condition

Supplemental Information

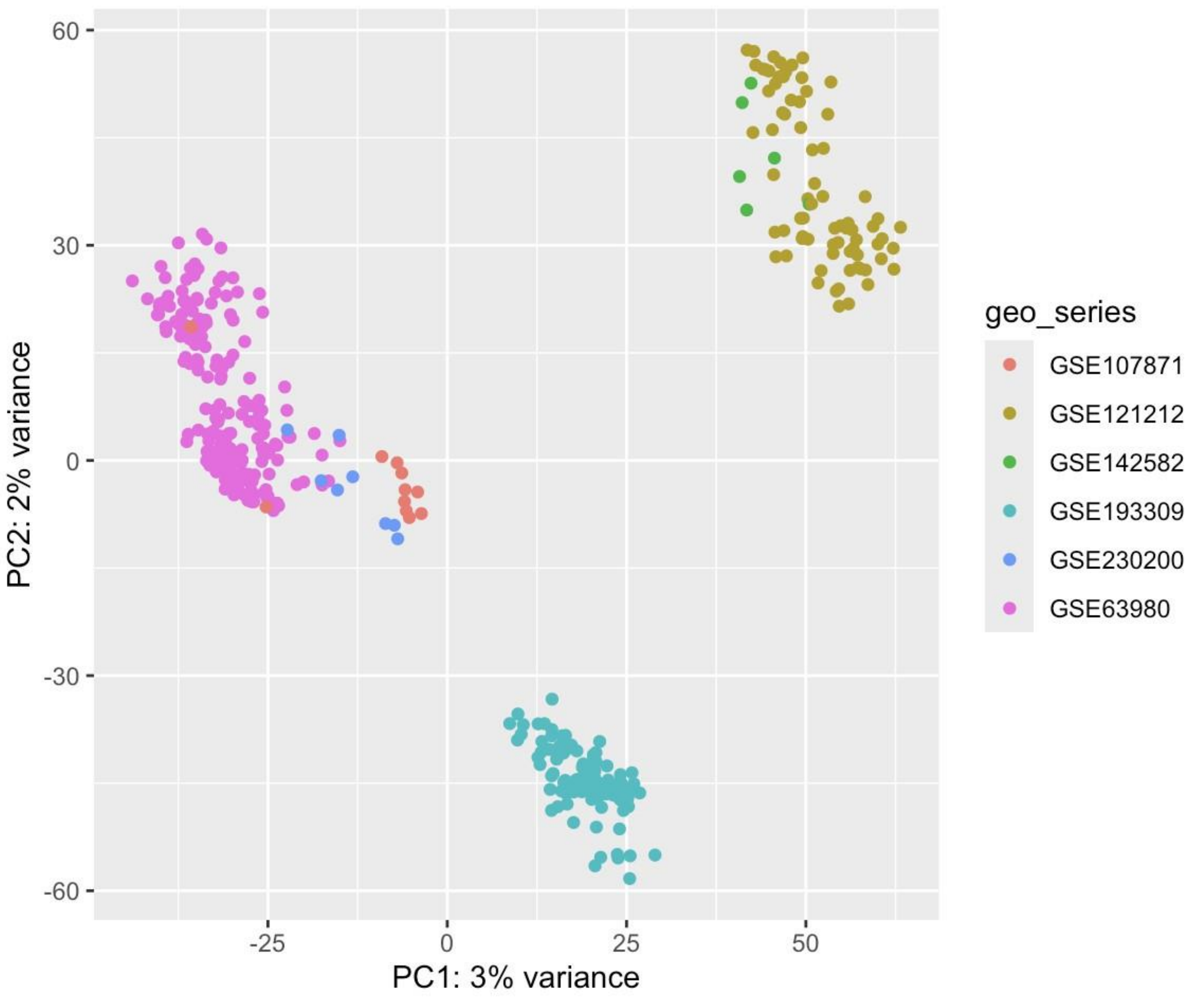

Figure S2: Principal component analysis (PCA) plot for GEO series

Supplemental Information

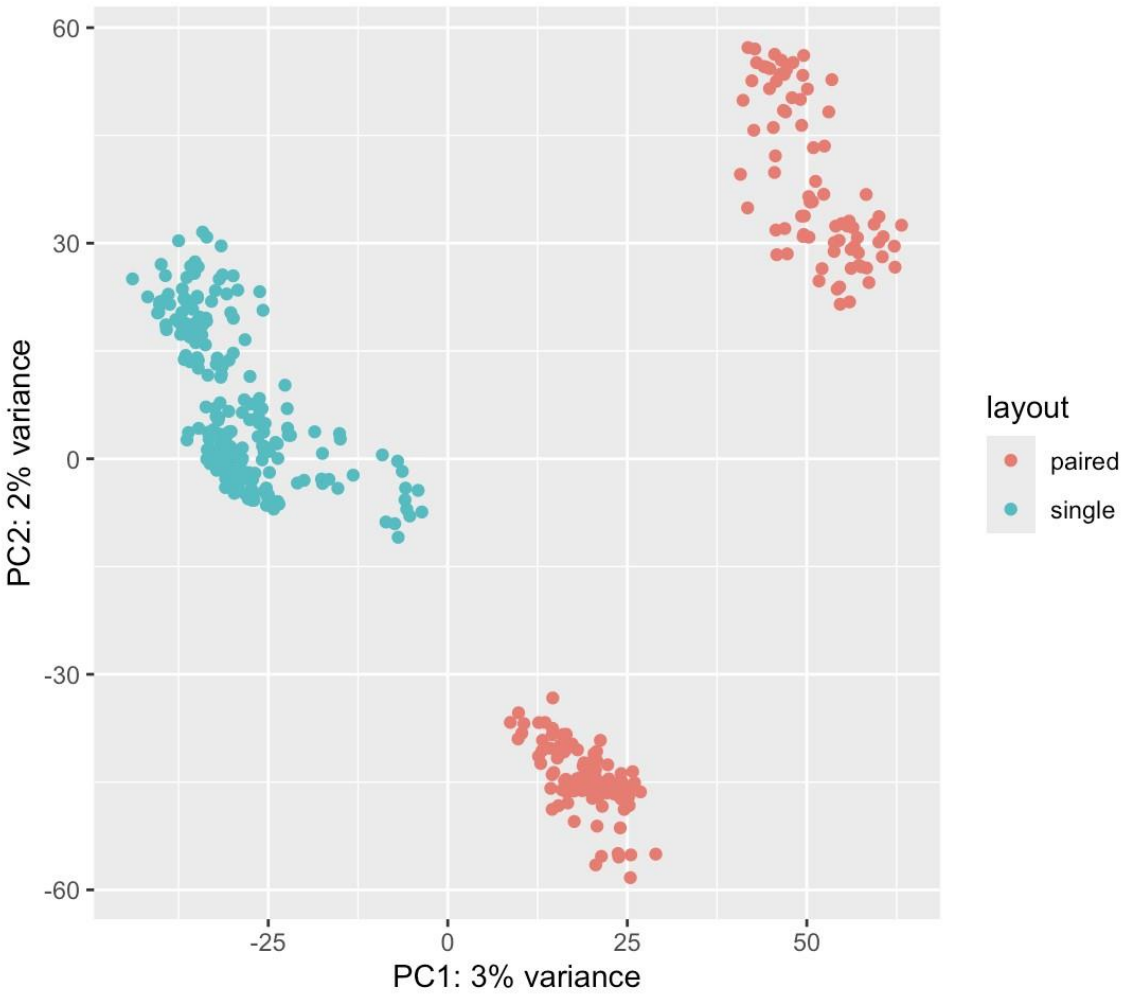

Figure S3: Principal component analysis (PCA) plot for single and paired end sequencing

Supplemental Information

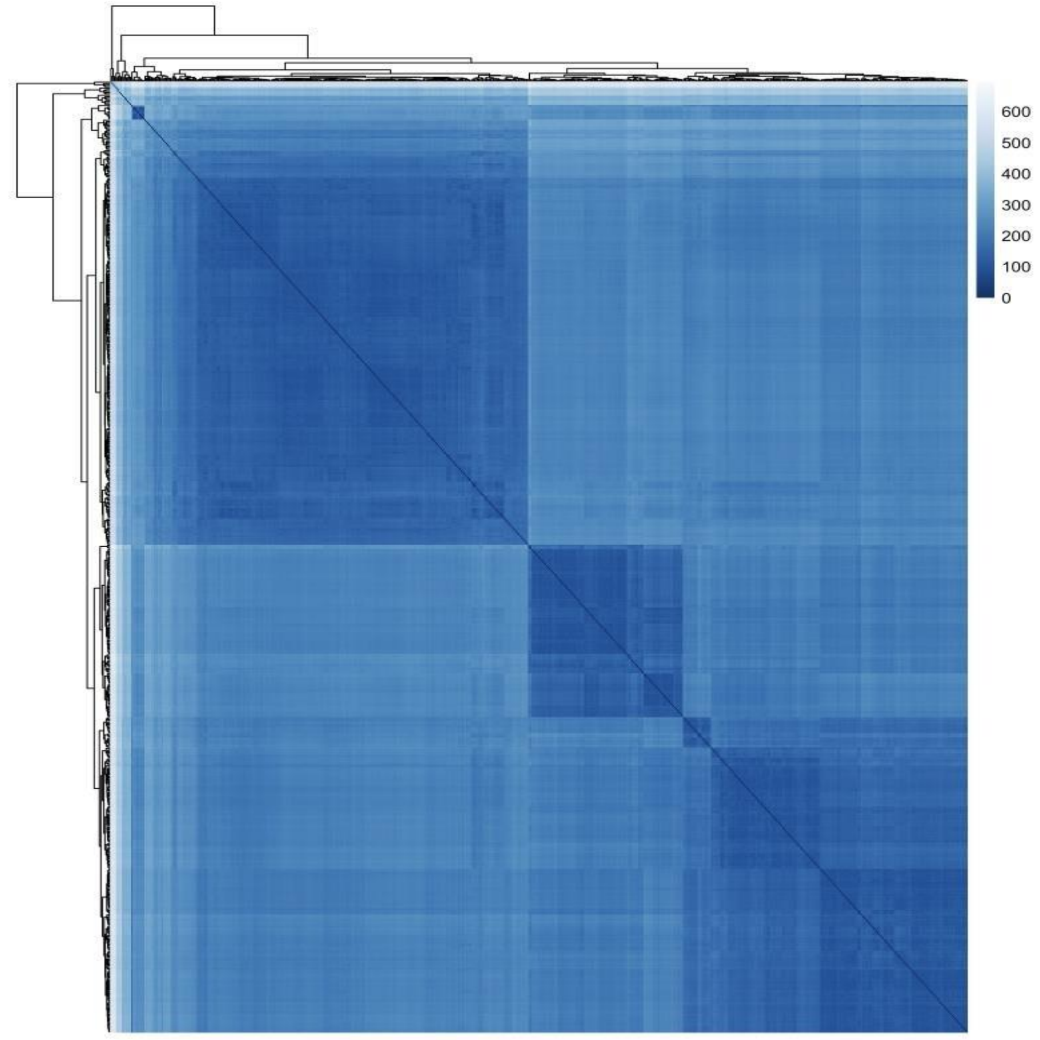

Figure S4: Hierarchical clustering between variance stabilized normalized log count values for all transcripts

Supplemental Information

Table S1: Significant gene of interest fold change in psoriatic lesional skin compared to healthy control

Gene	log2FoldChange	p-value	Differential expression
**GPR12**	-2.71847	8.19E-39	DOWN
**PPARG**	-1.05143	9.99E-10	DOWN
**TRPV4**	-1.07828	8.75E-36	DOWN
**HTR1A**	-0.86169	1.31E-2	DOWN
**PPARA**	-0.70541	1.31E-15	DOWN
**CNR2/CB2R**	0.661387	2.24E-05	UP
**TRPA1**	1.29784	2.07E-09	UP
**TRPV3**	1.384079	2.92E-30	UP
**PPARD**	1.434537	4.96E-61	UP
**GPR18**	1.636201	9.03E-25	UP
**HTR3B**	3.130148	1.70E-32	UP
**HTR3A**	4.081222	1.25E-85	UP
**ADORA2A**	0.76974	6.63E-04	UP

Supplemental Information

Table S2: Significant gene of interest fold change of psoriatic lesional skin compared to non-lesional skin

Gene	log2FoldChange	p-value	Differential expression
**PPARG**	-0.59669	2.84E-03	DOWN
**TRPV4**	-1.09205	4.84E-24	DOWN
**GPR12**	-2.36025	6.80E-20	DOWN
**PPARA**	-0.58724	7.51E-08	DOWN
**TRPV3**	1.177607	4.29E-15	UP
**PPARD**	1.341717	7.70E-35	UP
**GPR18**	1.359293	1.27E-11	UP
**TRPA1**	1.256991	3.51E-06	UP
**HTR3B**	2.674244	1.43E-16	UP
**HTR3A**	3.334127	4.18E-38	UP
**CNR2/CB2R**	0.739257	1.60E-04	UP
**ADORA2A**	0.815613	2.49E-03	UP

Supplemental Information

Table S3: Significantly upregulated/downregulated cannabinoid receptors and their potential role in psoriasis pathogenesis

**Receptor**	**Lesional to healthy**	**Lesional to non-lesional**	**Potential Role in Psoriasis**
**CNR2**	UP	UP	Regulating the inflammatory response[30]
**GPR12**	DOWN	DOWN	Unknown
**GPR18**	UP	UP	
**ADORA2A**	UP	UP	Regulating the adaptive and innate immune response[30]
**PPARG**	DOWN	DOWN	Regulating inflammation, lipid metabolism, and keratinocyte differentiation[19]
**PPARA**	DOWN	DOWN	
**PPARD**	UP	UP	Epithelial differentiation and wound healing.[27]
**HTR1A**	DOWN	-	Modulating the extracellular matrix[21]
**HTR3A**	UP	UP	Proliferation of keratinocytes, epidermal cell turnover,[32] psychological stress.[33]
**HTR3B**	UP	UP	
**TRPV4**	DOWN	DOWN	Skin barrier integrity, homeostasis, differentiation, nociception, and pruritis[17]
**TRPV3**	UP	UP	Pruritis[34], inflammation[34]
**TRPA1**	UP	UP	Keratinocyte differentiation, proliferation, and skin barrier function[35]
